# Involvement of placental growth factor upregulated *via* TGF-β1-ALK1-Smad1/5 signaling in prohaptoglobin-induced angiogenesis

**DOI:** 10.1371/journal.pone.0216289

**Published:** 2019-04-29

**Authors:** Mi-Kyung Oh, In-Sook Kim

**Affiliations:** 1 Department of Medical Life Sciences, College of Medicine, The Catholic University of Korea, Seoul, Republic of Korea; 2 Department of Biomedicine & Health Sciences, College of Medicine, The Catholic University of Korea, Seoul, Republic of Korea; Medical College of Wisconsin, UNITED STATES

## Abstract

A potential role of haptoglobin in arterial restructuring has been suggested, and our previous study demonstrated that prohaptoglobin, the precursor of haptoglobin, stimulates endothelial angiogenesis. However, the mechanisms underlying the angiogenic effects of prohaptoglobin are still unclear. Here, we investigated angiogenic signaling induced by prohaptoglobin using human umbilical vein endothelial cells. Prohaptoglobin upregulated the expression of placental growth factor (PlGF), vascular endothelial growth factor (VEGF)-A, and VEGF receptor 1 and 2, and also induced cell migration and tube network formation. PlGF knockdown attenuated these angiogenic effects of prohaptoglobin. Furthermore, a transcription factor profiling assay indicated that Smad is involved in PlGF expression in response to prohaptoglobin. Transforming growth factor-β1 (TGF-β1) expression and Smad1/5 phosphorylation were also induced by prohaptoglobin treatment. Blockade of TGF-β1 signaling using the TGF-β receptor kinase inhibitor LY2109761 or Smad1/5 siRNA reduced the prohaptoglobin-induced PlGF expression and *in vitro* tube formation. Knockdown of the TGF-β receptor ALK1, but not ALK5, with a specific siRNA blocked the Smad1/5 phosphorylation and PlGF expression induced by prohaptoglobin. These findings suggest that the angiogenic effects of prohaptoglobin are dependent on PlGF and mediated *via* a TGF-β1-ALK1-Smad1/5–PlGF/VEGFR1–VEGF-A/VEGFR2 signaling pathway.

## Introduction

New blood vessel formation occurs during many physiological and pathological processes, including embryonic development, wound healing, tissue regeneration, arthritis, and cancer growth [1]. Vascular endothelial growth factors (VEGFs) and their receptors are pivotal regulators of vascular formation. Mammals express five VEGF isoforms [VEGF-A, -B, -C, and -D, and placental growth factor (PlGF)] and three VEGF receptors (VEGFR1, 2, and 3). VEGF-A binds to VEGFR1 and 2, VEGF-B and PlGF bind exclusively to VEGFR1, and VEGF-C and -D bind to VEGFR2 and 3. The binding of VEGFs to VEGFRs induces tyrosine phosphorylation at specific sites on the receptors and triggers signaling cascades that culminate in neovascularization, angiogenesis, and lymphangiogenesis [2]. VEGF-A and VEGFR2 are the key angiogenic factors that stimulate endothelial proliferation, permeability, and migration, while VEGF-A/VEGFR1 signaling only weakly promotes angiogenic activity.

PlGF was originally identified in the placenta [3], but it has also been found to be expressed in endothelial cells, hematopoietic cells, tumor cells, and fibroblast-like cells [4, 5]. In vascular and non-vascular cells, PlGF participates in a wide range of pathological processes by activating vascular cells, protecting tumor cells from anti-cancer drugs, suppressing dendritic cells, and recruiting macrophages [5]. In particular, the binding of PlGF to VEGFR1 blocks VEGF-A from binding to this receptor and consequently enhances its binding to VEGFR2, which results in a more potent angiogenic signal. PlGF binding to VEGFR1 also results in the trans-phosphorylation of VEGFR2, which amplifies VEGF-A-driven angiogenesis [6]. Furthermore, PlGF is reported to upregulate VEGF-A expression and stimulate vascularization in ischemic tissues [7]. Therefore, PlGF is considered a pro-angiogenic factor that augments VEGFR2 signaling both directly and indirectly.

Transforming growth factor-β (TGF-β) is a multifunctional cytokine with roles in diverse biological processes such as cell growth, differentiation, inflammation, and angiogenesis [8, 9]. Signal transduction of TGF-β is mediated *via* transmembrane TGF-β receptor complexes consisting of type I and type II receptors with serine/threonine kinase activity. Association of the ligand to the heteromeric receptor complexes sequentially triggers the phosphorylation of the type II receptor and the type I receptor, which activates downstream transcription factors known as Smads and consequently induces the expression of TGF-β target genes [9]. Activin-like receptor kinase (ALK) 1 and ALK5 are two distinct type І receptors for TGF-β. ALK1 and ALK5 phosphorylate Smad1/5/8 and Smad2/3, respectively. The pro-angiogenic function of TGF-β in endothelial cells is mediated *via* an ALK1-containing receptor complex, while activation of ALK5-containing receptors inhibits angiogenesis [10, 11]. Thus, TGF-β may regulate the activation of the endothelium by regulating the balance between ALK1-Smad1/5/8 and ALK5-Smad2/3 signaling.

Haptoglobin (Hp) is a major acute-phase protein, and its plasma concentration increases in response to inflammation, infection, and various cancers [12, 13]. An important biological function of Hp is the clearance of free hemoglobin (Hb) from the circulation by forming a Hp–Hb complex that is removed by the macrophage scavenger receptor CD163 [14, 15], thus protecting tissues from Hb-mediated oxidative damage [16]. In addition, Hp has been suggested to play a role in vascular restructuring and the formation of new blood vessels [17–22]. Hp was upregulated in rabbit arteries after a sustained blood flow change, and the arterial Hp expression was dependent on nitric oxide synthesis [17, 18]. Increased Hp expression was detected in the myocardial interstitial fluid of animals in which coronary collaterals were induced by repetitive occlusion [19]. Hp also showed angiogenic and cell migrating properties [20, 21]. Previously, we reported that Hp enhanced the angiogenic potential of endothelial progenitor cells and restored blood perfusion after ischemic injury [22]. However, the underlying mechanisms through which Hp regulates angiogenesis are still unclear.

Hp is synthesized as a precursor form, prohaptoglobin (proHp), which is secreted into the circulation after proHp is processed to mature Hp in the endoplasmic reticulum [23]. However, we detected proHp in the sera of patients with liver cancer [24]. The biological significance of proHp secretion as the non-processed form and its function are unknown. Interestingly, our previous study demonstrated that proHp increased the expression of VEGF-A and VEGFR2 in endothelial cells and promoted tube formation, cell migration, and endothelial sprouting [24]. In the current study, we investigated the upstream signaling of proHp-induced angiogenesis and identified the involvement of PlGF and TGF-β1 signaling in proHp function.

## Materials and methods

### Cell culture and preparation of proHp-containing conditioned medium

Human umbilical vein endothelial cells (HUVECs; Lonza, Walkersville, MD, USA) were cultured in complete Endothelial Growth Medium (EGM-2 Bullet Kit; Clonetics, San Diego, CA, USA) supplemented with 5% FBS (Gibco/ThermoFisher Scientific, Waltham, MA, USA). HUVECs at passages 3–7 were used for this experiment.

COS-7 cells (American Type Culture Collection, Manassas, VA, USA) were maintained in DMEM (WelGENE, Daegu, Korea) supplemented with 10% FBS. *Hp2* cDNA was transfected into the COS-7 cells using Lipofectamine 2000 (Invitrogen/ThermoFisher Scientific), and the proHp-overexpressing cells were incubated in serum-free medium for 48 h [24]. The conditioned medium (CM) was collected, centrifuged at 3,000 rpm for 5 min, and filtered through a 0.22 μm Spin-X centrifuge tube filter (Costar, Corning, NY, USA). After the proHp concentration in the CM was determined by enzyme-linked immunosorbent assay (ELISA), the CM was used as a source of proHp. In experiments to characterize proHp activity, the proHp-containing COS-7 CM (proHp CM) was added into the HUVEC culture medium to a final proHp concentration of 0.2 μg/ml. Control CM was prepared by the same method as proHp CM except that it was collected from COS-7 cells transfected with an empty vector.

### Enzyme-linked immunosorbent assay (ELISA)

The proHp concentration in the CM was determined by sandwich ELISA, as described previously [24]. Briefly, CM samples containing proHp were added to ELISA plates coated with an anti-human Hp antibody (Sigma-Aldrich, H8638, St. Louis, MO, USA), and incubated for 2 h at room temperature. Captured proHp was incubated with an anti-human Hp secondary antibody conjugated to horseradish peroxidase (Abcam, Cambridge, UK). The assay was developed with tetramethylbenzidine substrate solution (GenDEPOT, Barker, TX, USA), and absorbance was measured at 450 nm using a Victor3 plate reader (PerkinElmer, Boston, MA, USA). As a standard, purified human Hp (2–2 type, Sigma-Aldrich) was used.

The levels of VEGF, PlGF, and TGF-β1 in culture media from proHp-stimulated HUVECs were measured using ELISA kits (R&D Systems, Minneapolis, MN, USA) according to the manufacturer’s instructions. For TGF-β1 measurement, prior to running the assay, latent TGF-β1 was activated by adding 1 M HCl to the cell culture medium, followed by neutralization with 1.2 M NaOH/0.5 M HEPES, according to the manufacturer’s protocol. Absorbance for each assay was measured at 450 nm using a Victor3 plate reader, and the results were normalized to the total protein content, which was determined by colorimetric Bradford protein assay (Bio-Rad, Hercules, CA, USA).

### Reverse-transcription PCR (RT-PCR)

Total RNA was extracted using RNA STAT-60 (Tel-test Inc., Gainesville, FL, USA), and 2 μg of total RNA was reverse transcribed into cDNA for 1 h at 42°C using M-MLV reverse transcriptase (Promega, Madison, WI, USA). The cDNA aliquots were amplified on a Mycyler Thermal Cycler (Bio-Rad) using Go Tag DNA polymerase (Promega) and the gene primers listed in Table 1. Each PCR cycle consisted of 94°C for 1 min, 60°C for 1 min, and 72°C for 1 min. The PCR products were loaded onto a 1% agarose gel containing 0.01% (v/v) SafeView nucleic acid dye (Applied Biological Materials Inc., Richmond, Canada), electrophoresed, and photographed under a UV light using the Gel Doc XR+ Gel Documentation System (Bio-Rad). Gene-specific primers (Table 1) were designed using Primer3 software (http://primer3.ut.ee/) and NCBI Primer-BLAST (https://www.ncbi.nlm.nih.gov/tools/primer-blast/).

**Table 1:** Oligonucleotide primers used for gene expression analysis.

Gene	Primer sequence	Size (bp)
VEGF-A	5′-GCGGAGAAAGCATTTGTTTGT-3′ 5′-CGGCTTGTCACATCTGCAA-3′	124
VEGF-B	5′-AGCACCAAGTCCGGATG-3′ 5′-GTCTGGCTTCACAGCACTG-3′	128
VEGF-C	5′-CACGAGCTACCTCAGCAAGA-3′ 5′-GCTGCCTGACACTGTGGTA-3′	186
VEGF-D	5′-CCTGAAGAAGATCGCTGTTC-3′ 5′-GAGAGCTGGTTCCTGGAGAT-3′	144
PlGF	5′-GTTCAGCCCATCCTGTGTCT-3′ 5′-AACGTGCTGAGAGAACGTCA-3′	136
VEGFR1	5′-AGCAAGTGGGAGTTTGC-3′ 5′-AGGTCCCGATGAATGC-3′	617
VEGFR2	5′-TGGGAACCGGAACCTCACTATC-3′ 5′-GTCTTTTCCTGGGCACCTTCTATT-3′	132
VEGFR3	5′-CAACAGACCCACACAGAACT-3′ 5′-TTTCCATCCTTGTACCACTG-3′	263
TGF-β1	5′-CAGAAATACAGCAACAATTC-3′ 5′-TTGCAGTGTGTTATCCCTGC-3′	186
ALK1	5′-ACAACATCCTAGGCTTCATCGCCT-3′ 5′-TGGTTTGCCCTGTGTACCGAAGAT-3	212
ALK5	5′-CGTTACAGTGTTTCTGCCACCT-3′ 5′-AGACGAAGCACACTGGTCCAGC-3′	315
GAPDH	5′-ACCACAGTCCATGCCATCAC-3′ 5′-TCCACCACCCTGTTGCTGTA-3′	452

### Western blotting and immunoprecipitation

Cell lysates were electrophoresed on SDS-polyacrylamide gels and transferred onto nitrocellulose blotting membranes (GE Healthcare, Freiburg, Germany). Blots were probed with monoclonal anti-Smad1, anti-Smad2/3, anti-phospho-Smad1/5, or anti-phospho-Smad2/3 antibodies (Cell Signaling Technologies, Danvers, MA, USA), followed by a horseradish peroxidase-conjugated secondary antibody (Sigma-Aldrich). Signals were detected using a chemiluminescence detection kit (Amersham Biosciences, Buckinghamshire, UK) and a luminescent image analyzer (LAS-3000; Fujifilm, Tokyo, Japan).

To evaluate VEGF receptor phosphorylation, HUVEC extracts (400 μg of protein) were mixed with 4 μg of VEGFR1 or VEGFR2 polyclonal antibodies (Santa Cruz Biotechnology, Santa Cruz, CA, USA) and Protein G-agarose beads (Invitrogen/ThermoFisher Scientific), and incubated at 4°C overnight with gentle rotation. Immune complexes were precipitated by centrifugation and analyzed by western blotting using an anti-phospho-tyrosine antibody (Santa Cruz Biotechnology).

### RNA interference

To silence the expression of PlGF, ALK1, and ALK5, HUVECs were transiently transfected with specific siRNAs consisting of a mixture of four oligonucleotides targeting different regions of the same gene (ON-TARGETplus SMARTpool, Dharmacon, Chicago, IL, USA) using DharmaFECT4 reagent (Dharmacon). After incubating each siRNA (25 nM final concentration) with DharmaFECT 4 reagent for 20 min at room temperature, the mixture was applied to HUVEC cultures and the cells were incubated for 24 h at 37°C. Silencing of Smad1/5 was performed by transfection with a mixture of Smad1 siRNA (12.5 nM final concentration) and Smad5 siRNA (12.5 nM final concentration) (ON-TARGETplus SMARTpool, Dharmacon) using DharmaFECT4 transfection reagent. As a negative control, cells were transfected with a non-specific siRNA pool (siCONTROL Non-Targeting siRNA pool, Dharmacon) at a final concentration of 25 nM. Gene silencing was verified by RT-PCR or western blot analysis.

### *In vitro* tube formation assay

HUVECs were seeded in 48-well plates (4 × 10^4^ cells/well) precoated with growth factor-reduced Matrigel (BD Biosciences, San Jose, CA, USA), and proHp CM or control CM was added to the media to a final proHp concentration of 0.2 μg/ml [24]. After proHp exposure for 6 h, tube formation was observed under an inverted microscope, and the total tube length was measured in three randomly chosen fields using ImageJ software (National Institutes of Health).

### Migration assay

For cell migration assays, Transwell inserts (24-well) with an 8.0 μm pore size (Costar, Corning) were used. In the upper chamber of the Transwell, 5 × 10^4^ cells were seeded in serum-free Endothelial Basal Medium (EBM-2; Clonetics) and the same medium with control CM or proHp CM (final proHp concentration: 0.2 μg/ml) was added to the lower chamber. After incubation for 24 h at 37°C, non-migrating cells on the upper surface of the insert were removed with a cotton swab, and the migrating cells were fixed in cold 100% methanol for 20 min and stained with 1% crystal violet for 20 min. Images of the migrating cells were taken using a microscope. The migrating cells were also quantified by detecting the absorbance at 595 nm after the stain was extracted with 10% acetic acid for 30 sec.

### Transcription factor profiling assay

HUVECs were treated with proHp CM (final proHp: 0.2 μg/ml), and incubated for 14 h. Nuclear proteins from the cells were extracted with the Nuclear Extraction Kit (Signosis, Santa Clara, CA, USA), and transcription factor activation was detected using the Transcription Factor Activation Array І (Signosis), according to the manufacturer’s instructions. Briefly, the nuclear extracts were incubated with the probe mix for 30 min at room temperature, and then the transcription factor/probe complexes were bound to a spin column. The bound probes were separated from the complexes with elution buffer and hybridized to plates precoated with DNA sequences that are complementary to the specific probes. The captured probes were detected with streptavidin-HRP. The luminescence was measured on a Victor3 luminometer (PerkinElmer, Boston, MA, USA) and expressed as relative light units (RLUs).

### Immunodepletion assay

ProHp CM, containing 1 μg of proHp, was incubated with 3 μg of polyclonal antibody against Hp (Sigma-Aldrich, H8638) for 4 h at 4°C with gentle rotation. As a negative control, proHp CM was incubated with the equivalent amount of normal rabbit IgG (Santa Cruz Biotechnology, sc-2027). After the incubation, 30 μl of Protein G-agarose beads (50% slurry, Invitrogen/ThermoFisher Scientific, 15920010) was added, and the mixtures were further incubated at 4°C overnight. The immune complexes were pelleted by centrifugation at 14,000 rpm for 1 min at 4°C, and the supernatants were collected. ProHp depletion in the CM was confirmed by western blot analysis.

### Statistical analysis

Statistical analysis was performed using PASW Statistics software (version 18; SPSS, Chicago, IL, USA). Results were expressed as the mean ± SD. Normality tests confirmed that the data were distributed normally, so statistical analyses were performed by Student’s t-test or one-way analysis of variance (ANOVA) followed by Tukey’s post-test analysis. A p-value of less than 0.05 was considered statistically significant.

## Results

### Regulation of the expression of VEGF and VEGFR family members by proHp

Since *Hp*-transfected COS-7 cells secreted proHp into the cell culture medium, we used conditioned medium (CM) from the proHp-overexpressing COS-7 cells (proHp CM) as a source of proHp in all experiments to investigate the angiogenic activity of proHp. After the proHp concentration in the CM was determined by ELISA, HUVECs were treated with the CM to a final proHp concentration of 0.2 μg/ml in the cell culture medium. As a control, a similar volume of control CM from empty vector-transfected COS-7 cells was added to the HUVEC cultures.

**Fig 1:**
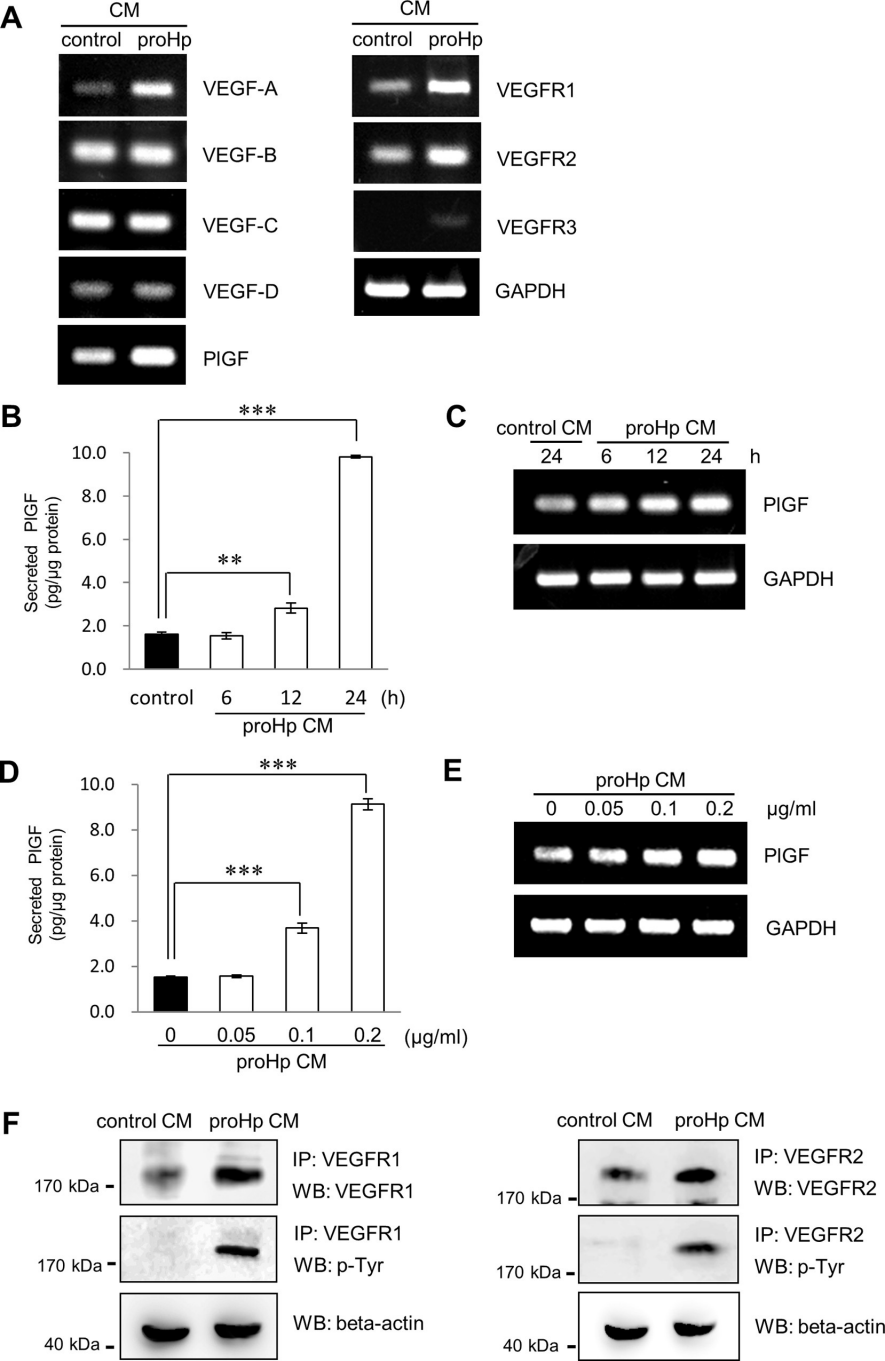
Effects of proHp on the expression of VEGF and VEGFR family members. (A) HUVECs were incubated in 1% FBS-supplemented EBM-2 medium containing proHp CM (final proHp concentration in culture medium: 0.2 μg/ml) or a similar volume of control CM. After 24 h of incubation, mRNA levels of VEGF and VEGFR family members were measured by RT-PCR. (B and C) HUVECs were incubated with proHp CM (final proHp concentration: 0.2 μg/ml) for 6, 12, or 24 h. At the indicated times, the levels of secreted PlGF protein and mRNA were determined by ELISA and RT-PCR, respectively. (D and E) PlGF protein and mRNA were analyzed in HUVECs treated for 24 h with proHp CM (final proHp concentration: 0.05–0.2 μg/ml). The ELISA results are expressed as the mean ± SD of three independent experiments. **P < 0.01 and ***P < 0.001 vs. control CM-treated cells (one-way ANOVA). (F) Tyrosine phosphorylation of VEGFR1 and VEGFR2 in proHp CM (final proHp concentration: 0.2 μg/ml)-treated HUVECs was detected by immunoprecipitation/western blotting. All figures are representative of independent experiments performed at least twice.

To identify the effects of proHp on the expression of VEGF family members and their receptors, HUVECs were incubated with proHp CM or control CM for 24 h. As shown in Fig 1A, treatment with proHp CM increased the expression of PlGF and VEGFR1 as well as VEGF-A and VEGFR2 compared with the control CM treatment. When HUVECs were incubated with proHp CM for different times or with various concentrations of proHp, PlGF expression was increased by proHp CM treatment in a time- and concentration-dependent manner at both the protein and the mRNA level (Fig 1B–1E). ProHp CM also induced the expression of VEGFR1 and VEGFR2 (Fig 1F). These findings indicate that not only VEGF-A/VEGFR2 signaling, but also PlGF/VEGFR1 signaling, may be involved in proHp-induced angiogenesis.

### The induction of PlGF-dependent angiogenesis by proHp

**Fig 2:**
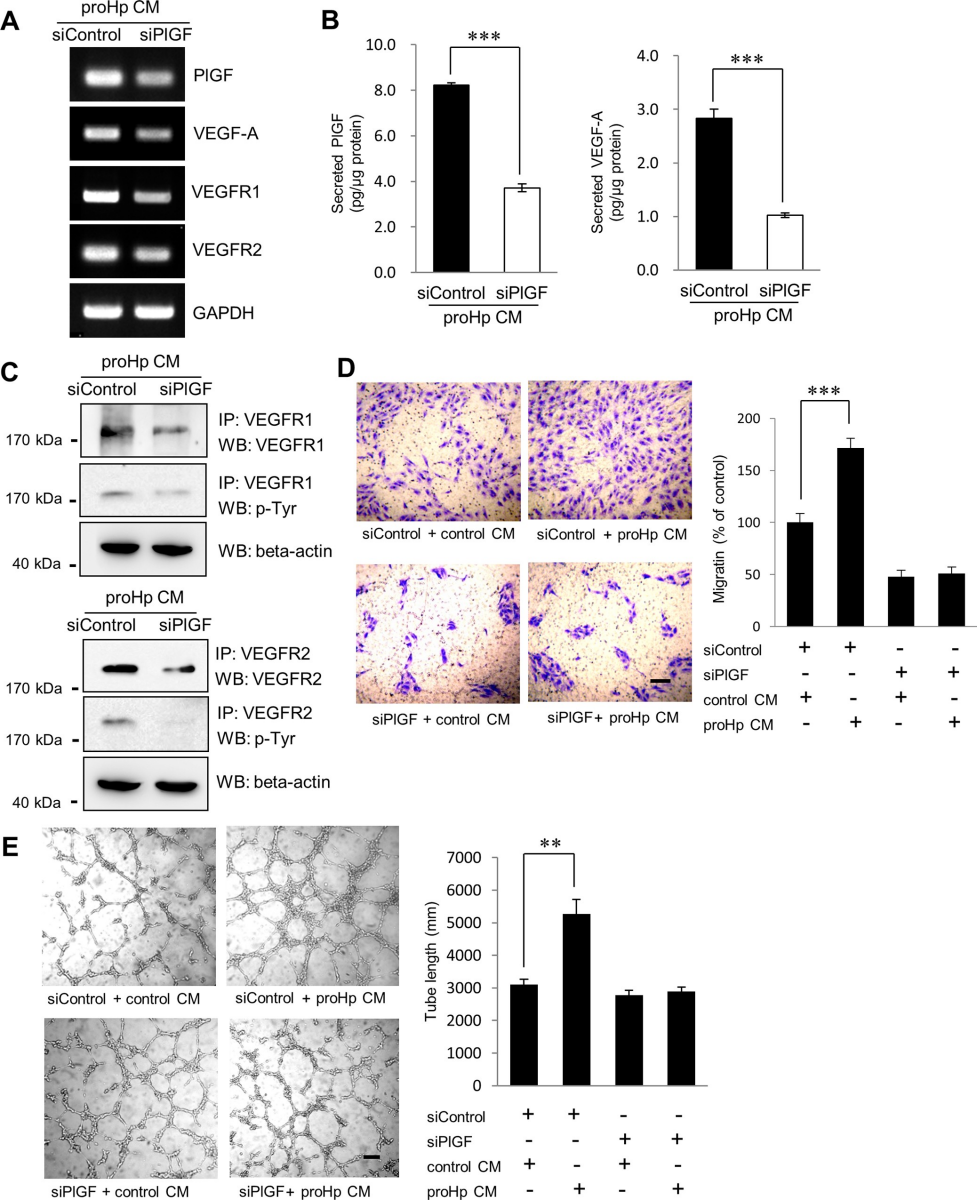
Attenuation of proHp-induced angiogenesis by PlGF knockdown. HUVECs were transfected with control siRNA (siControl) or PlGF-specific siRNA (siPlGF) and then incubated for 24 h in 1% FBS-supplemented EBM-2 medium containing proHp CM (final 0.2 μg/ml proHp). (A) The expression of PlGF, VEGF-A, VEGFR1, and VEGFR2 was determined by RT-PCR. (B) Secreted PlGF and VEGF-A were measured by ELISA. The results represent the mean ± SD of three independent experiments. ***P < 0.001 vs. control siRNA-transfected cells (Student’s t-test). (C) Tyrosine phosphorylation of VEGFR1 and VEGFR2 was analyzed by immunoprecipitation/western blotting. (D) siRNA-transfected cells were seeded in the upper chambers of Transwell inserts, and serum-free EBM-2 media containing control CM or proHp CM (final 0.2 μg/ml proHp) was added to the lower chambers. After incubation for 24 h at 37°C, non-migrating cells were removed with a cotton swab, and the migrating cells were fixed in cold 100% methanol for 20 min and stained with 1% crystal violet for 20 min. The stained cells were observed under a microscope (scale bar = 100 μm) and quantified by measuring the absorbance at 595 nm after the stain was extracted with 10% acetic acid for 30 sec. ***P < 0.001 vs. control CM-treated cells (Student’s t-test). (E) siRNA-transfected cells grown on growth factor-reduced Matrigel were incubated for 6 h in medium with proHp CM (final 0.2 μg/ml proHp) or control CM. The formation of tube networks was observed, and total tube lengths were measured in three randomly chosen fields (scale bar = 100 μm). **P < 0.01 vs. control CM-treated cells (Student’s t-test). Data shown in (A-E) are representative of independent experiments repeated twice.

HUVECs were transfected with PlGF-specific siRNA and then treated with proHp CM. siRNA-mediated knockdown of PlGF significantly decreased the proHp-induced expression of VEGF-A, VEGFR1, and VEGFR2 (Fig 2A and 2B). Tyrosine phosphorylation of VEGFR1 and VEGFR2 was also reduced by PlGF knockdown (Fig 2C). Moreover, the stimulating effects of proHp CM on cell migration and tube network formation were not observed when HUVECs were transfected with PlGF siRNA (Fig 2D and 2E). These results suggest that the angiogenic activity of proHp CM on HUVECs is PlGF-dependent.

### The role of Smad1/5 on PlGF expression and proHp-induced angiogenesis

**Fig 3:**
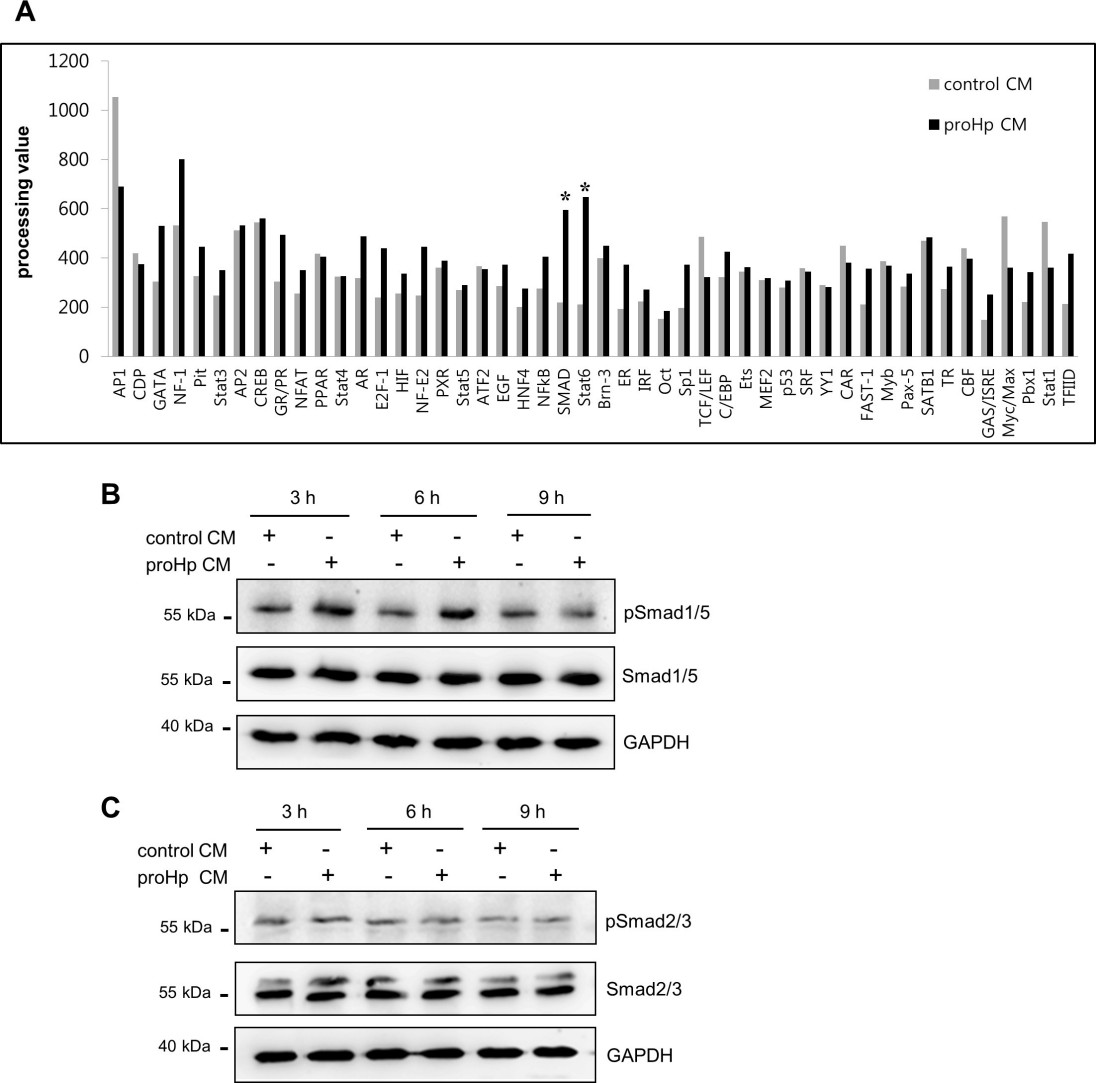
Smad as proHp-inducible transcription factors. (A) HUVECs were incubated for 14 h with proHp CM (final 0.2 μg/ml proHp) or control CM in serum-free EBM-2 medium. Nuclear fractions were extracted and subjected to a transcription factor profiling assay as described in the Materials and methods. (B and C) After 3 h of serum starvation, HUVECs were stimulated with proHp CM (final 0.2 μg/ml proHp) or control CM in serum-free EBM-2 medium. At the indicated times, phospho-Smad1/5 (B) and phospho-Smad2/3 (C) in cell lysates were detected by western blot.

To identify the transcription factors regulating PlGF expression in HUVECs, a transcription factor activation profiling assay was performed using nuclear extracts from cells treated with proHp CM or control CM. Particularly high transcriptional activity of Smad was detected in proHp-treated cells compared with control cells (Fig 3A). The phosphorylation of Smad1/5 was also increased by treatment of HUVECs with proHp CM, but little change was observed in Smad2/3 phosphorylation (Fig 3B and 3C).

**Fig 4:**
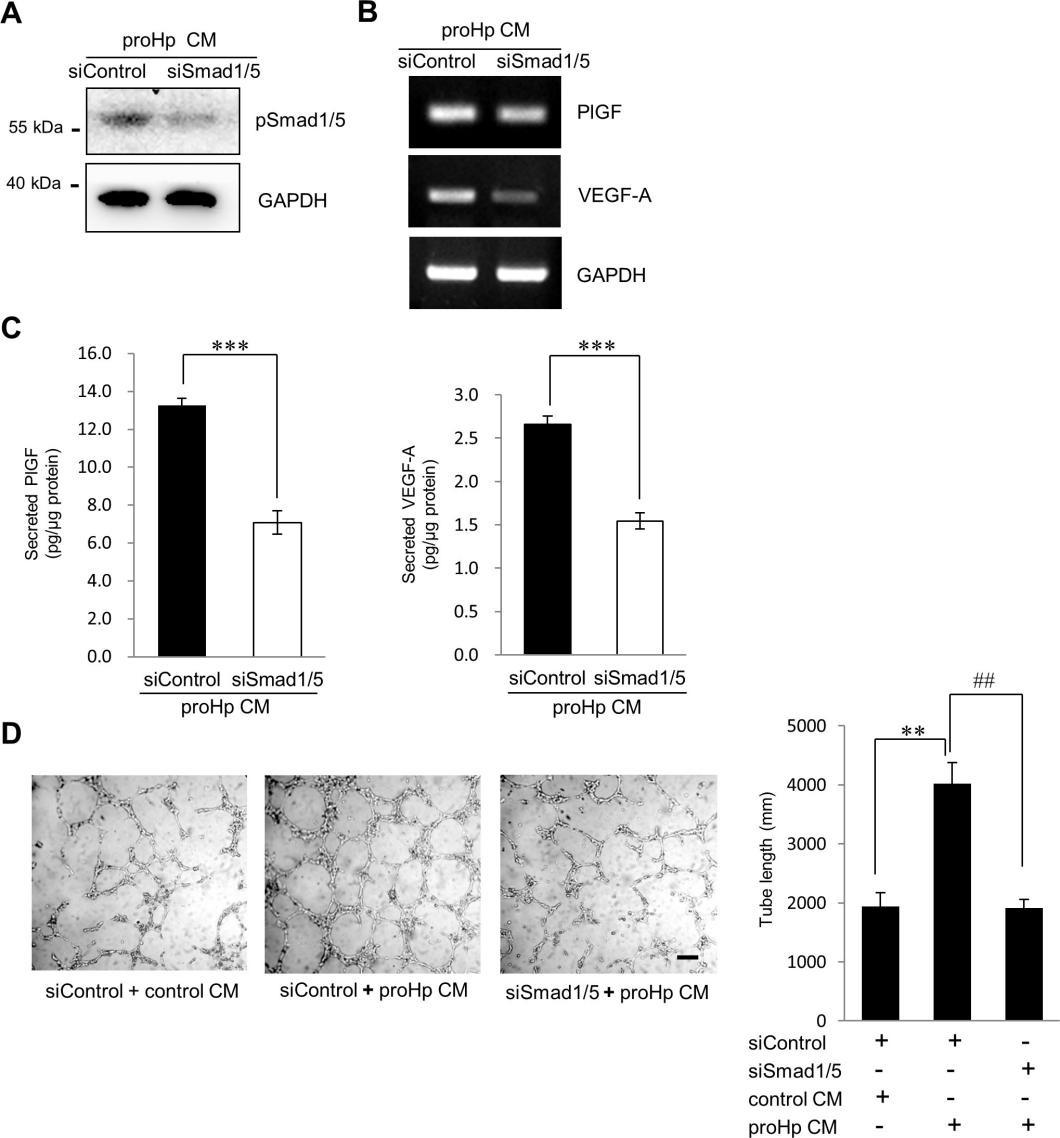
Requirement of Smad1/5 activity for proHp-induced PlGF expression and angiogenesis. HUVECs were transfected with control siRNA (siControl) or Smad1/5 siRNA (siSmad1/5). (A) After 24 h, the cells were incubated in serum-free EBM-2 medium for 3 h and further incubated with proHp CM (final 0.2 μg/ml proHp) for 6 h. A reduction in phospho-Smad1/5 following stimulation with proHp CM was confirmed by western blot. (B and C) After transfection with siRNA, the cells were incubated for 24 h in EBM-2 medium containing 1% FBS and proHp CM (final 0.2 μg/ml proHp). The mRNA and protein levels of PlGF and VEGF-A were analyzed in the Smad1/5 knockdown cells. ***P < 0.001 vs. control siRNA-transfected cells (Student’s t-test). (D) The Smad1/5 knockdown cells were seeded onto Matrigel and incubated for 6 h with proHp CM (final 0.2 μg/ml proHp) or control CM. Total tube lengths in three randomly chosen fields were quantitated. Results are expressed as the mean ± SD. **P < 0.01 vs. control CM-treated cells. ^##^P < 0.01 vs. control siRNA-transfected cells (Student’s t-test). Experiments were performed at least twice with similar results, and representative data are shown here.

To examine whether Smad1/5 is critical for proHp-induced, PlGF-dependent angiogenesis, Smad1/5 was knocked down in HUVECs with specific siRNA. Knockdown of Smad1/5 attenuated the proHp-induced expression of PlGF and VEGF-A at both the mRNA and protein levels (Fig 4A–4C). Tube formation was also inhibited by Smad1/5 knockdown (Fig 4D). On the other hand, STAT6 knockdown had no effect on proHp-induced PlGF or VEGF-A expression (S1 Fig), even though the transcriptional activity of STAT6 was also higher in proHp-treated HUVECs than in control cells (Fig 3A). Taken together, these data indicate that Smad1/5 is a significant transcription factor driving the expression of PlGF, which is critical for proHp-induced endothelial angiogenesis.

### Involvement of TGF-β1/ALK1/Smad1/5 signaling in proHp-induced, PlGF-dependent angiogenesis

**Fig 5:**
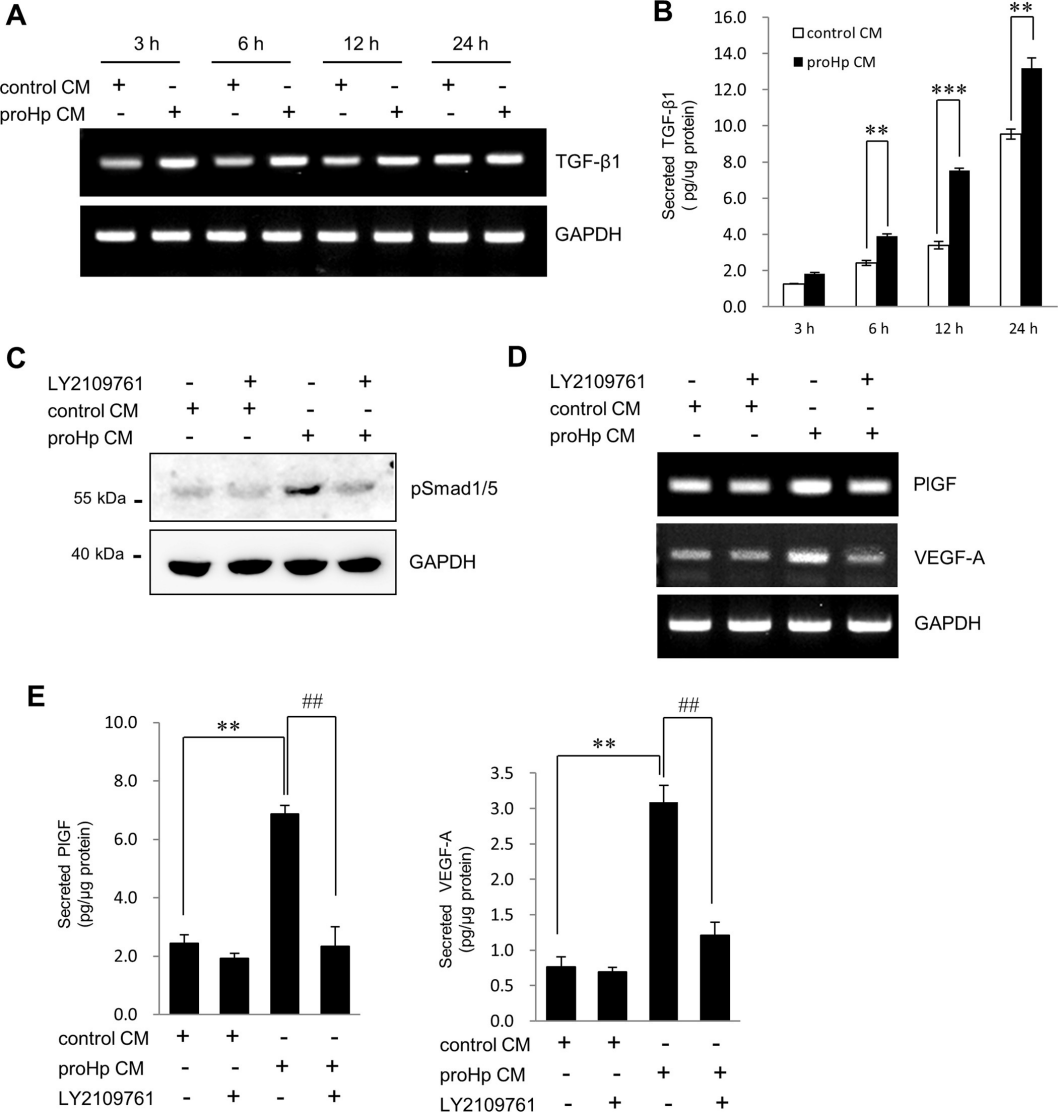
Involvement of TGF-β1 signaling in proHp-induced PlGF and VEGF-A expression. (A and B) After HUVECs were serum-starved for 3 h, the cells were treated with control CM or proHp CM (final 0.2 μg/ml proHp) for the indicated times. TGF-β1 mRNA levels and secreted protein levels were determined by RT-PCR and ELISA, respectively. **P < 0.01 and ***P < 0.001 vs. control CM-treated cells (Student’s t-test). (C) After serum starvation for 3 h, the cells were preincubated for 1 h in the presence or absence of the TGFβRІ/ІІ kinase inhibitor LY2109761 (1 μM). The cells were then further incubated for 6 h in EBM-2 medium containing proHp CM (final 0.2 μg/ml proHp). Smad1/5 phosphorylation was analyzed by western blot. (D and E) After pretreatment with the inhibitor LY2109761 for 1 h, the cells were incubated for 24 h in EBM-2 medium containing 1% FBS and proHp CM (final 0.2 μg/ml proHp). The expression of PlGF and VEGF-A was determined, and the data are shown as the mean ± SD of three independent experiments. **P < 0.01 vs. control CM-treated cells, ^##^P < 0.01 vs. no inhibitor treatment (Student’s t-test).

To confirm whether TGF-β induction is associated with the Smad1/5 activation, TGF-β1 expression was analyzed in HUVECs treated with proHp CM. The levels of TGF-β1 mRNA and protein were significantly increased at 6 h after proHp treatment (Fig 5A and 5B). Pretreatment with an inhibitor of the TGF-β receptor kinase LY2109761 effectively blocked the stimulatory effects of proHp CM on PlGF and VEGF-A expression and Smad1/5 phosphorylation (Fig 5C–5E).

**Fig 6:**
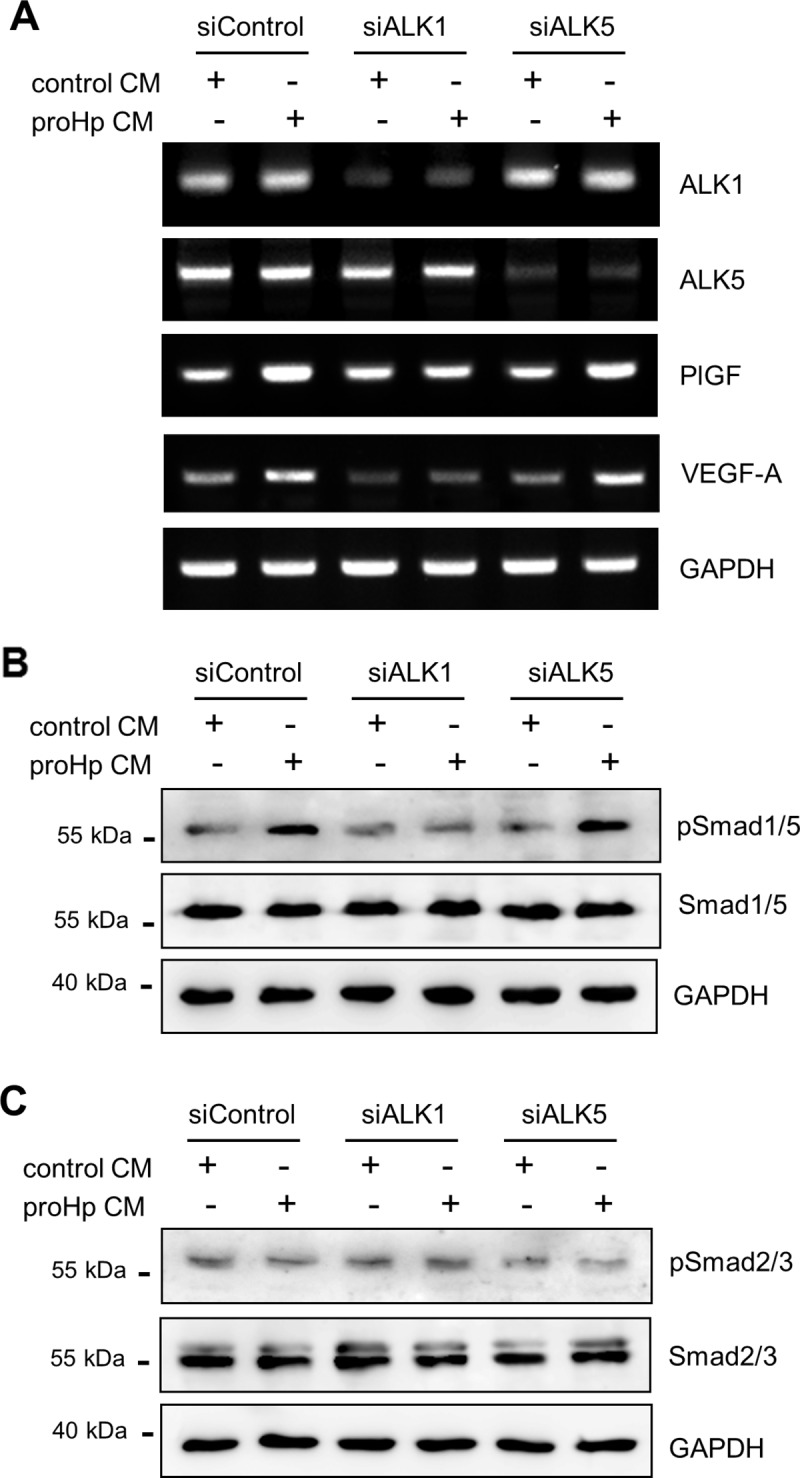
ALK1-mediated Smad1/5 phosphorylation, and PlGF and VEGF-A expression. HUVECs were transfected with a control siRNA (siControl) or siRNAs against ALK1 (siALK1) or ALK5 (siALK5). (A) After 24 h, the cells were incubated in EBM-2 medium containing 1% FBS and proHp CM (final 0.2 μg/ml proHp) for 24 h. mRNA levels of angiogenic regulators were analyzed by RT-PCR. (B and C) After transfection with siRNA for 24 h, the cells were incubated in serum-free EBM-2 medium for 3 h and further incubated with proHp CM (final 0.2 μg/ml proHp) for 6 h. Phosphorylation of Smads was determined by western blot. All experiments were performed at least twice with similar results, and representative data are shown here.

To establish the involvement of the ALK1- or ALK5-containing TGF-β receptor, these receptors were knocked down in HUVECs with specific siRNAs. In the ALK1-depleted cells, Smad1/5 phosphorylation and expressions of PlGF and VEGF-A were not increased by proHp CM treatment (Fig 6A and 6B). However, knockdown of ALK5 had no effects on proHp-enhanced gene expressions of PlGF and VEGF-A and Smad1/5 phosphorylation in HUVECs (Fig 6A and 6B). Smad2/3 phosphorylation was not changed by ALK1 knockdown and was only modestly decreased by ALK5 knockdown, and proHp had no effect on Smad2/3 phosphorylation in ALK1- or ALK5-knockdown HUVECs (Fig 6C). These findings suggest that the angiogenic function of proHp CM is mediated *via* the TGF-β1/ALK1/Smad1/5 signaling pathway.

**Fig 7:**
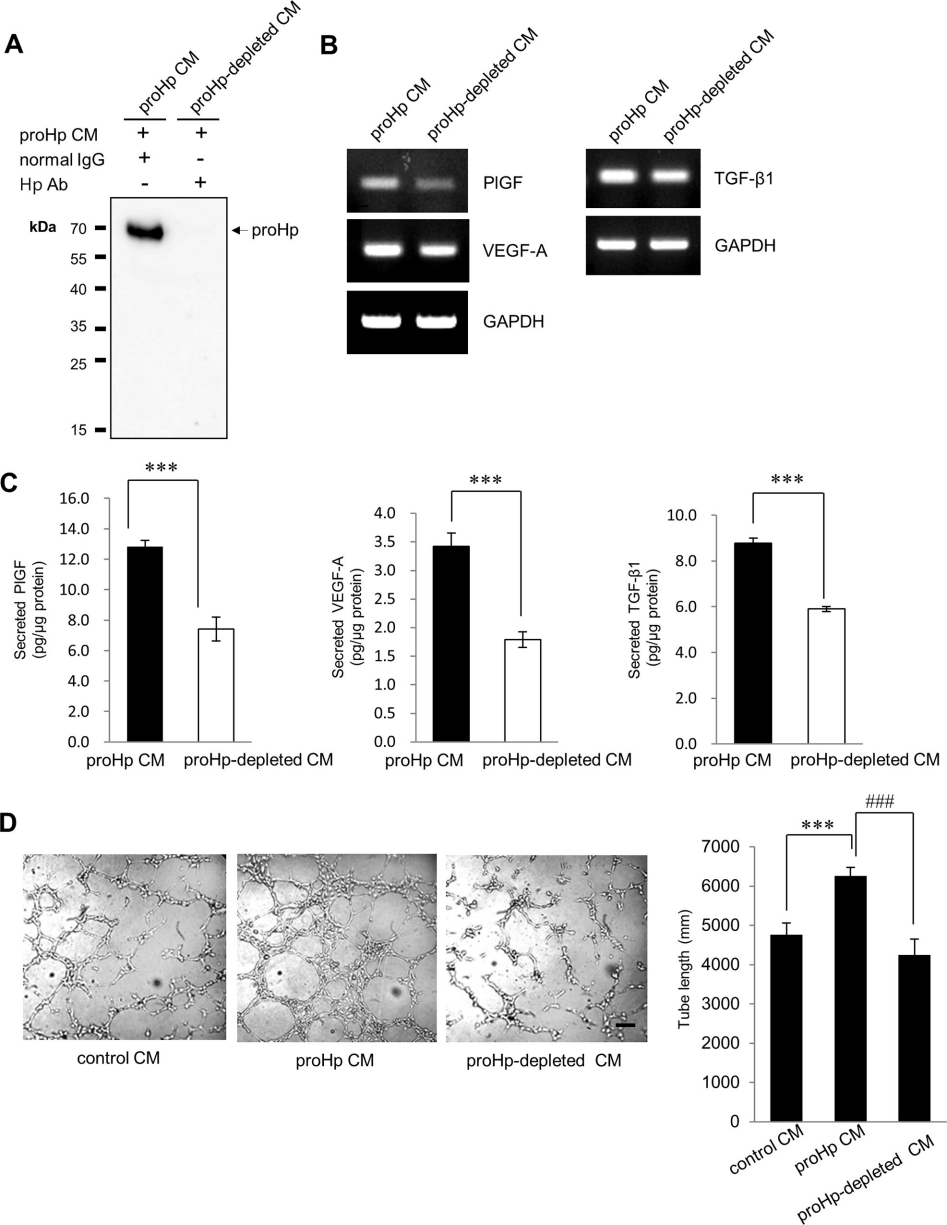
Inhibition of the angiogenic effects of COS-7 CM by depleting proHp. (A) The CM obtained from proHp-overexpressing COS-7 cells was depleted of proHp with an Hp antibody as described in the Materials and methods, and proHp depletion in the CM was confirmed by western blot. (B and C) HUVECs were treated with the proHp-depleted CM or mock-depleted CM (proHp CM; incubated with rabbit IgG instead of an Hp antibody), and the expression of angiogenic factors was analyzed by RT-PCR and ELISA after 6 h (for TGF-β1) or 24 h (for PlGF and VEGF-A). ***P < 0.001 vs. cells treated with proHp CM (Student’s t-test). (D) HUVECs on Matrigel were incubated in mock-depleted CM (proHp CM) or proHp-depleted CM. After 6 h, tube lengths were calculated in three randomly chosen fields. ***P < 0.001 vs. cells treated with control IgG-treated CM, ^###^P < 0.001 vs. proHp CM-treated cells (Student’s t-test). Data shown in (A-D) are representative of independent experiments repeated twice.

To verify that these angiogenic effects were due to proHp and not caused by other factors in CM obtained from cultures of proHp-overexpressing COS-7 cells, antibody-mediated depletion of proHp from the CM was performed. As shown in Fig 7, elimination of proHp from the CM abrogated its ability to induce the expression of PlGF, VEGF-A, and TGF-β1 in HUVECs and its ability to stimulate endothelial tube formation. These data confirm that the angiogenic effects of the proHp-containing COS-7 CM in this study were due to the proHp in the CM.

## Discussion

**Fig 8:**
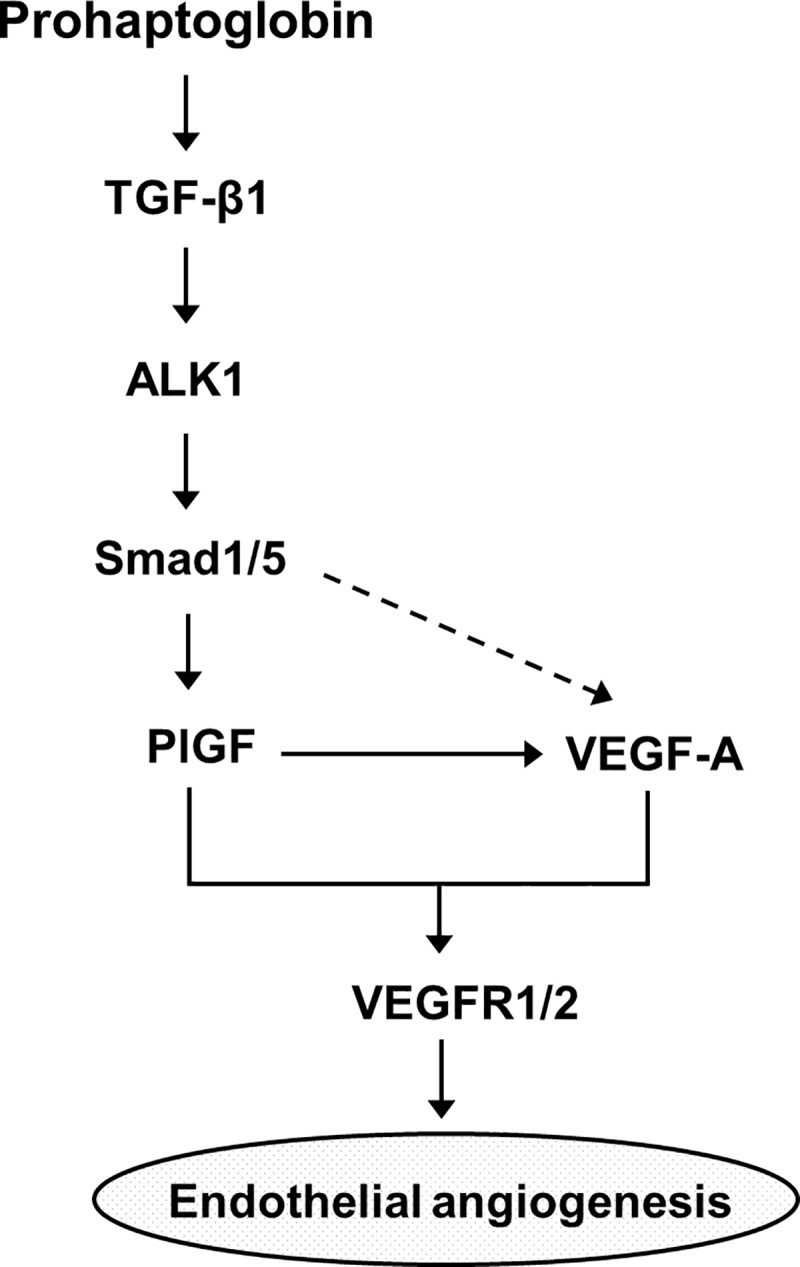
The proposed signaling pathways in proHp-induced angiogenesis.

In the present study, we provide evidence of the involvement of the TGF-β1-ALK1-Smad1/5 signaling pathway and PlGF in proHp-induced angiogenesis. In HUVECs, proHp upregulated PlGF (Fig 1A–1E), and PlGF knockdown inhibited proHp-induced angiogenic events (VEGF-A and VEGFR1/2 expression, cell migration, and *in vitro* tube formation) (Fig 2A–2E). In addition, proHp induced TGF-β1 expression (Fig 5A and 5B), and Smad1/5 activation through the TGF-β receptor ALK1 was associated with the proHp-induced PlGF expression (Fig 4A–4C and Fig 6A). These results strongly suggest that proHp promotes PlGF-dependent angiogenesis *via* TGF-β1 signaling. Based on these findings, we propose that proHp induces a TGF-β1-ALK1-Smad1/5–PlGF/VEGFR1–VEGF-A/VEGFR2 signaling cascade, resulting in angiogenic activity (Fig 8). To our knowledge, this is the first report to suggest the participation of TGF-β1 signaling and PlGF in proHp or Hp function.

TGF-β can exert both pro- and anti-angiogenic effects, and these opposing roles are likely dependent on different TGF-β receptors and phosphorylation of different downstream Smads. Smad1/5/8 phosphorylation *via* TGF-β/ALK1 contributes to angiogenesis, whereas Smad2/3 phosphorylation *via* TGF-β/ALK5 inhibits angiogenesis and leads to maturation of blood vessels [10]. In the present study, proHp increased Smad1/5 phosphorylation but had no effect on Smad2/3 phosphorylation (Fig 3B and 3C). Based on knockdown experiments using specific siRNAs, the proHp-induced Smad1/5 phosphorylation occurred through ALK1 but not ALK5 (Fig 6B and 6C). The expression level of each receptor and the presence of co-receptors determine which type I receptor, ALK1 or ALK5, is activated. In activated endothelial cells, both ALK1 and ALK5 are expressed, and endothelial cells also express a TGF-β accessory receptor, endoglin (CD105), which promotes endothelial angiogenesis by potentiating TGF-β/ALK1 signaling and negatively regulating TGF-β/ALK5 signaling [25]. To date, we do not know why proHp induces only TGF-β/ALK1 signaling even though TGF-β also has a high affinity for the receptor ALK5. One possibility is that proHp may affect the function of a regulatory co-receptor. Further studies will be required to determine the effect of proHp on endoglin activation in HUVECs. In addition, proHp increased TGF-β1 expression (Fig 5A and 5B), and inhibition of TGF-β1 signaling by siRNA-mediated knockdown of Smad1/5 or ALK1 attenuated the proHp-induced upregulation of PlGF and VEGF-A (Fig 4B and 4C, and Fig 6A). Taken together, these findings suggest that TGF-β1/ALK1 signaling is an upstream event involved in the induction of endothelial angiogenesis by proHp.

Our results show that PlGF contributes to proHp-induced angiogenesis, as PlGF knockdown reduced proHp-mediated angiogenic events including VEGF-A expression, VEGFR1 and R2 phosphorylation, and cell migration and tube formation. Other previous studies also showed that PlGF upregulates VEGF-A expression and stimulates angiogenesis and collateral vessel growth in ischemic tissues [7, 26]. Since binding of PlGF to VEGFR1 leads to intermolecular cross-talk between VEGFR1 and VEGFR2, resulting in amplification of VEGF-A/VEGFR2 signaling, PlGF is considered a positive regulator of VEGF-A-driven angiogenesis [6]. However, it was also reported that PlGF inhibited VEGF-A/VEGFR2 signaling by heterodimerizing with VEGF-A and blocking its binding to VEGFR2 [27]. The negative effect of PlGF on VEGFR2 signaling may be dependent on the level of PlGF being high enough to form a complex with VEGF-A. We do not know whether PlGF and VEGF-A dimerize or whether PlGF induces cooperative cross-talk between VEGFR1 and VEGFR2 under our experimental conditions. However, in a preliminary experiment, we observed increased expression of the PlGF co-receptor neurophilin (NRP)-1 in proHp-treated cells, in addition to the increase in PlGF, VEGF-A, VEGFR1, and VEGFR2. Therefore, it is possible that PlGF may bind to VEGFR1/NRP-1 and positively regulate VEGF-A/VEGFR2 signaling. In a further study, we will investigate the exact molecular role of PlGF in proHp-induced angiogenesis.

Cid *et al*. reported a pro-angiogenic function of Hp purified from human serum [20]. To date, we do not know which isoform of Hp or proHp is an active angiogenic molecule *in vivo*. The presence of proHp in the circulation has been overlooked, since most precursor proHp is processed to Hp intracellularly before secretion. However, we recently detected proHp in the sera of patients with liver cancer [24], suggesting that a small amount of proHp may exist in the sample of Hp isolated from patient serum. Follow-up studies will be necessary to compare the effects of proHp and mature Hp on angiogenesis and the related molecular mechanisms.

In summary, we identified for the first time TGF-β1, ALK1 and Smad1/5 as key upstream signaling molecules in proHp-promoted endothelial angiogenesis. The proHp-stimulated Smad1/5 activation induced PlGF and VEGF-A expressions, and led to angiogenesis. Our findings suggest that the angiogenic effect of proHp is dependent on PlGF and mediates *via* a TGF-β1-ALK1-Smad1/5–PlGF/VEGFR1–VEGF-A/VEGFR2 signaling pathway.

## Supporting information

S1 Fig No effects of STAT6 on the expression of PlGF and VEGF-A. (TIF)

## References

[1] Carmeliet P. Angiogenesis in health and disease. Nature medicine. 2003;9(6):653–60. Epub 2003/06/05. 10.1038/nm0603-653 . 12778163

[2] Olsson AK, Dimberg A, Kreuger J, Claesson-Welsh L. VEGF receptor signalling—in control of vascular function. Nature reviews Molecular cell biology. 2006;7(5):359–71. Epub 2006/04/25. 10.1038/nrm1911 . 16633338

[3] Maglione D, Guerriero V, Viglietto G, Delli-Bovi P, Persico MG. Isolation of a human placenta cDNA coding for a protein related to the vascular permeability factor. Proceedings of the National Academy of Sciences of the United States of America. 1991;88(20):9267–71. Epub 1991/10/15. 1924389 10.1073/pnas.88.20.9267 PMC52695

[4] De Falco S. The discovery of placenta growth factor and its biological activity. Experimental & molecular medicine. 2012;44(1):1–9. Epub 2012/01/10. 10.3858/emm.2012.44.1.025 22228176 PMC3277892

[5] Dewerchin M, Carmeliet P. PlGF: a multitasking cytokine with disease-restricted activity. Cold Spring Harbor perspectives in medicine. 2012;2(8). Epub 2012/08/22. 10.1101/cshperspect.a011056 22908198 PMC3405829

[6] Autiero M, Waltenberger J, Communi D, Kranz A, Moons L, Lambrechts D, et al Role of PlGF in the intra- and intermolecular cross talk between the VEGF receptors Flt1 and Flk1. Nature medicine. 2003;9(7):936–43. Epub 2003/06/11. 10.1038/nm884 . 12796773

[7] Carmeliet P, Moons L, Luttun A, Vincenti V, Compernolle V, De Mol M, et al Synergism between vascular endothelial growth factor and placental growth factor contributes to angiogenesis and plasma extravasation in pathological conditions. Nature medicine. 2001;7(5):575–83. Epub 2001/05/01. 10.1038/87904 . 11329059

[8] Pardali E, Ten Dijke P. TGFbeta signaling and cardiovascular diseases. International journal of biological sciences. 2012;8(2):195–213. Epub 2012/01/19. 10.7150/ijbs.3805 22253564 PMC3258560

[9] Goumans MJ, Liu Z, ten Dijke P. TGF-beta signaling in vascular biology and dysfunction. Cell research. 2009;19(1):116–27. Epub 2008/12/31. 10.1038/cr.2008.326 . 19114994

[10] Goumans MJ, Valdimarsdottir G, Itoh S, Rosendahl A, Sideras P, ten Dijke P. Balancing the activation state of the endothelium via two distinct TGF-beta type I receptors. The EMBO journal. 2002;21(7):1743–53. Epub 2002/04/03. 10.1093/emboj/21.7.1743 11927558 PMC125949

[11] Pardali E, Goumans MJ, ten Dijke P. Signaling by members of the TGF-beta family in vascular morphogenesis and disease. Trends in cell biology. 2010;20(9):556–67. Epub 2010/07/27. 10.1016/j.tcb.2010.06.006 . 20656490

[12] Levy AP, Asleh R, Blum S, Levy NS, Miller-Lotan R, Kalet-Litman S, et al Haptoglobin: basic and clinical aspects. Antioxidants & redox signaling. 2010;12(2):293–304. Epub 2009/08/08. 10.1089/ars.2009.2793 . 19659435

[13] Quaye IK. Haptoglobin, inflammation and disease. Transactions of the Royal Society of Tropical Medicine and Hygiene. 2008;102(8):735–42. Epub 2008/05/20. 10.1016/j.trstmh.2008.04.010 . 18486167

[14] Andersen CB, Torvund-Jensen M, Nielsen MJ, de Oliveira CL, Hersleth HP, Andersen NH, et al Structure of the haptoglobin-haemoglobin complex. Nature. 2012;489(7416):456–9. Epub 2012/08/28. 10.1038/nature11369 . 22922649

[15] Kristiansen M, Graversen JH, Jacobsen C, Sonne O, Hoffman HJ, Law SK, et al Identification of the haemoglobin scavenger receptor. Nature. 2001;409(6817):198–201. Epub 2001/02/24. 10.1038/35051594 . 11196644

[16] Lim YK, Jenner A, Ali AB, Wang Y, Hsu SI, Chong SM, et al Haptoglobin reduces renal oxidative DNA and tissue damage during phenylhydrazine-induced hemolysis. Kidney international. 2000;58(3):1033–44. Epub 2000/09/06. 10.1046/j.1523-1755.2000.00261.x . 10972668

[17] Smeets MB, Pasterkamp G, Lim SK, Velema E, van Middelaar B, de Kleijn DP. Nitric oxide synthesis is involved in arterial haptoglobin expression after sustained flow changes. FEBS letters. 2002;529(2–3):221–4. Epub 2002/10/10. . 12372604 10.1016/s0014-5793(02)03343-4

[18] Smeets MB, Sluijter JP, Donners MM, Velema E, Heeneman S, Pasterkamp G, et al Increased arterial expression of a glycosylated haptoglobin isoform after balloon dilation. Cardiovascular research. 2003;58(3):689–95. Epub 2003/06/12. . 12798443 10.1016/s0008-6363(03)00294-3

[19] Lohr NL, Warltier DC, Chilian WM, Weihrauch D. Haptoglobin expression and activity during coronary collateralization. American journal of physiology Heart and circulatory physiology. 2005;288(3):H1389–95. Epub 2004/11/20. 10.1152/ajpheart.00938.2004 . 15550518

[20] Cid MC, Grant DS, Hoffman GS, Auerbach R, Fauci AS, Kleinman HK. Identification of haptoglobin as an angiogenic factor in sera from patients with systemic vasculitis. The Journal of clinical investigation. 1993;91(3):977–85. Epub 1993/03/01. 10.1172/JCI116319 7680672 PMC288050

[21] de Kleijn DP, Smeets MB, Kemmeren PP, Lim SK, Van Middelaar BJ, Velema E, et al Acute-phase protein haptoglobin is a cell migration factor involved in arterial restructuring. FASEB journal: official publication of the Federation of American Societies for Experimental Biology. 2002;16(9):1123–5. Epub 2002/06/01. 10.1096/fj.02-0019fje . 12039846

[22] Park SJ, Baek SH, Oh MK, Choi SH, Park EH, Kim NH, et al Enhancement of angiogenic and vasculogenic potential of endothelial progenitor cells by haptoglobin. FEBS letters. 2009;583(19):3235–40. Epub 2009/09/16. 10.1016/j.febslet.2009.09.014 . 19751729

[23] Wicher KB, Fries E. Prohaptoglobin is proteolytically cleaved in the endoplasmic reticulum by the complement C1r-like protein. Proceedings of the National Academy of Sciences of the United States of America. 2004;101(40):14390–5. Epub 2004/09/24. 10.1073/pnas.0405692101 15385675 PMC521962

[24] Oh MK, Park HJ, Lee JH, Bae HM, Kim IS. Single chain precursor prohaptoglobin promotes angiogenesis by upregulating expression of vascular endothelial growth factor (VEGF) and VEGF receptor2. FEBS letters. 2015;589(9):1009–17. Epub 2015/03/18. 10.1016/j.febslet.2015.03.006 . 25775978

[25] Lebrin F, Goumans MJ, Jonker L, Carvalho RL, Valdimarsdottir G, Thorikay M, et al Endoglin promotes endothelial cell proliferation and TGF-beta/ALK1 signal transduction. The EMBO journal. 2004;23(20):4018–28. Epub 2004/09/24. 10.1038/sj.emboj.7600386 15385967 PMC524335

[26] Luttun A, Tjwa M, Moons L, Wu Y, Angelillo-Scherrer A, Liao F, et al Revascularization of ischemic tissues by PlGF treatment, and inhibition of tumor angiogenesis, arthritis and atherosclerosis by anti-Flt1. Nature medicine. 2002;8(8):831–40. Epub 2002/07/02. 10.1038/nm731 . 12091877

[27] Eriksson A, Cao R, Pawliuk R, Berg SM, Tsang M, Zhou D, et al Placenta growth factor-1 antagonizes VEGF-induced angiogenesis and tumor growth by the formation of functionally inactive PlGF-1/VEGF heterodimers. Cancer cell. 2002;1(1):99–108. Epub 2002/06/28. . 12086892 10.1016/s1535-6108(02)00028-4

