# Supplementary figures and images for: Involvement of placental growth factor upregulated via TGF-β1-ALK1-Smad1/5 signaling in prohaptoglobin-induced angiogenesis
Source: PLoS One. 2019 Apr 29;14(4):e0216289. doi: 10.1371/journal.pone.0216289 (PMC6488081; doi:10.1371/journal.pone.0216289)

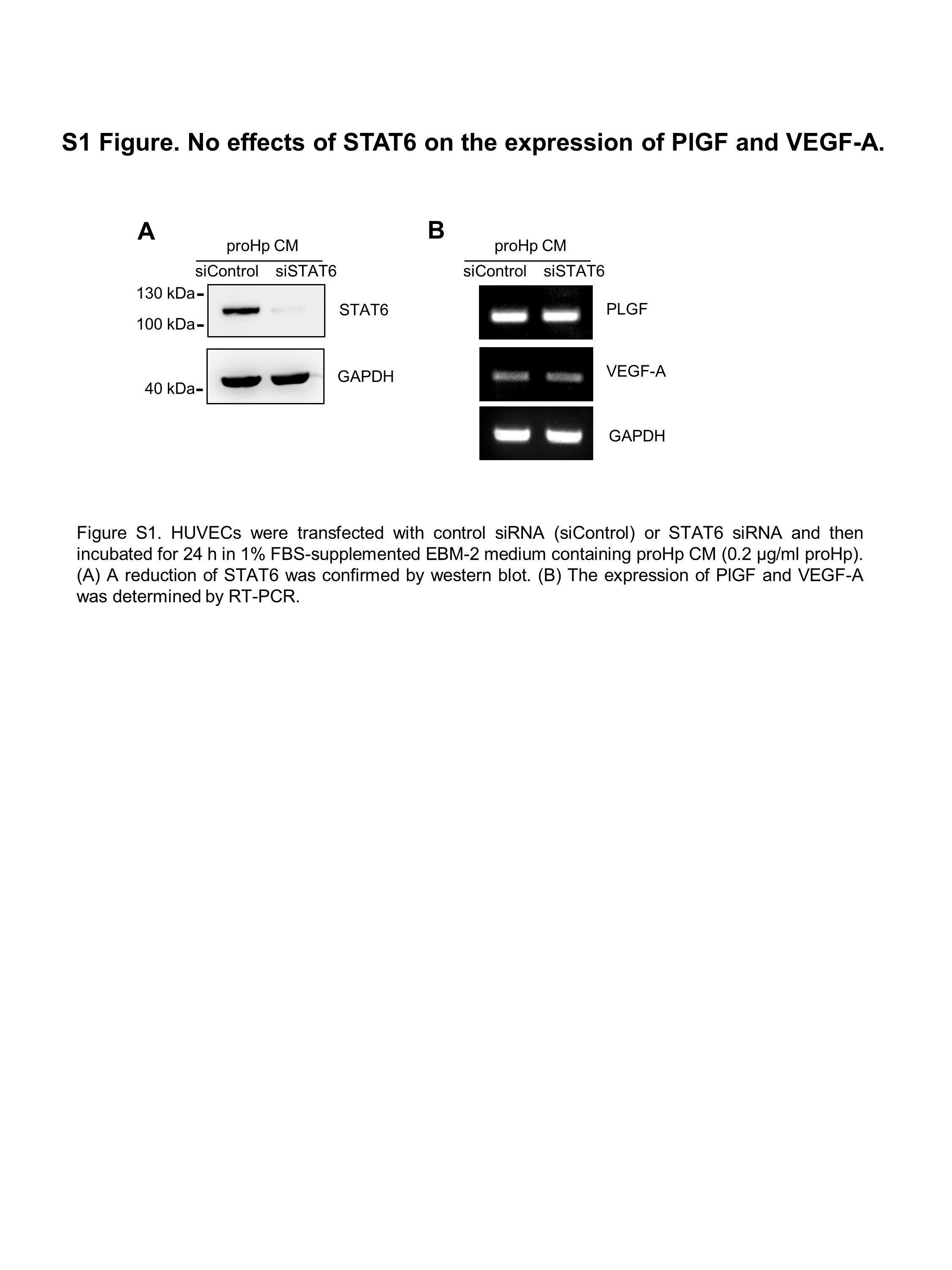

Supplement: S1 Fig — (TIF) [file pone.0216289.s001.TIF]
